# Measurement of Force and Intramuscular Pressure Changes Related to Thrust Spinal Manipulation in an In Vivo Animal Model

**DOI:** 10.3390/biology12010062

**Published:** 2022-12-30

**Authors:** William R. Reed, Carla R. Lima, Michael A. K. Liebschner, Christopher P. Hurt, Peng Li, Maruti R. Gudavalli

**Affiliations:** 1 Department of Physical Therapy, University of Alabama at Birmingham, Birmingham, AL 35294, USA; 2 Rehabilitation Sciences Program, University of Alabama at Birmingham, Birmingham, AL 35294, USA; 3 Department of Medicine, Baylor College of Medicine, Houston, TX 77030, USA; 4 School of Nursing, University of Alabama at Birmingham, Birmingham, AL 35294, USA; 5 College of Chiropractic Medicine, Keiser University, West Palm Beach, FL 33411, USA

**Keywords:** spinal manipulation, intramuscular, viscoelasticity, biomechanics, force, multifidus, muscle, manual therapy, lumbar, spine

## Abstract

**Simple Summary:**

Spinal manipulation is recommended by clinical guidelines to treat low back pain. However, our understanding of how spinal manipulation alleviates pain and knowledge of the role that viscoelastic tissue properties play in the dampening of applied spinal manipulation forces in vivo is limited. Superficial and deep intramuscular pressures and applied forces were measured using miniature pressure catheters and a force transducer during low back spinal manipulation using a clinically available spinal manipulation device (Activator V) or a feedback motor in a deeply anesthetized animal model. Viscoelastic properties of muscle and other soft tissues greatly diminished applied spinal manipulative forces as well as intramuscular pressures in deep spinal tissues. These animal model findings may eventually have clinical implications or help to determine how spinal manipulation helps to alleviate muscular low back pain, particularly if it is determined that certain thresholds of deep tissue forces or intramuscular pressure changes are required to initiate a cascade of biological mechanisms resulting in a positive clinical treatment response.

**Abstract:**

Current knowledge regarding biomechanical in vivo deep tissue measures related to spinal manipulation remain somewhat limited. More in vivo animal studies are needed to better understand the effects viscoelastic tissue properties (i.e., dampening) have on applied spinal manipulation forces. This new knowledge may eventually help to determine whether positive clinical outcomes are associated with particular force thresholds reaching superficial and/or deep spinal tissues. A computer-controlled feedback motor and a modified Activator V device with a dynamic load cell attached were used to deliver thrust spinal manipulations at various magnitudes to the L7 spinous process in deeply anesthetized animals. Miniature pressure catheters (Millar SPR-1000) were inserted unilaterally into superficial and deep multifidi muscles. Measurements of applied mechanical forces and superficial/deep multifidi intramuscular pressure changes were recorded during spinal manipulations delivered in vivo. Manipulative forces and net changes in intramuscular pressures reaching deep spinal tissues are greatly diminished by viscoelastic properties of in vivo tissues, which could have possible clinical safety and/or mechanistic implications.

## 1. Introduction

In a study involving 195 countries, low back pain (LBP) was the leading cause of lost productivity and years lived with disability [1]. Identifying the anatomical sources of LBP is diagnostically challenging, however paraspinal muscle and surrounding soft tissues are often a primary focus in clinical evaluation prior to delivery of manual therapy treatment. Spinal manipulation (high velocity low amplitude thrusts) are currently recommended by a majority of clinical guidelines as a conservative, non-surgical treatment for LBP [2,3,4]. Spinal manipulation involves the delivery of short duration mechanical thrusts applied to the spine using either direct hand contact (<150 ms thrust duration) or commercially available mechanical devices such as the Activator V device (≤5 ms thrust duration) [5,6,7,8]. Spinal manual therapy (spinal manipulation and spinal mobilization) forces have been shown to result in appreciable loads passing through a cadaveric disc [9,10,11]. Most of our current knowledge pertaining to the biomechanical-related effects of manual therapy on deep spinal tissues is associated with cadaveric studies [9,10,11,12,13,14,15,16,17]. However, inherent limitations of cadaveric and/or in vitro spinal manipulation studies include extensive disruption or removal of trunk tissue, manipulative forces being delivered directly to an isolated or potted spine, swelling of spinal tissues, loss of muscular or reflex response, and/or experimentally induced alterations in force dampening effects related to the viscoelastic properties of surrounding tissues; which combined likely skew biomechanical-related data compared to when spinal manipulation is delivered in vivo [11,14,18,19].

In vivo animal studies investigating physiological effects of spinal manipulation dosage are few [20]. Colloca et al. examined neurophysiological [21] (electromyographic activation and compound action potentials) and biomechanical [22] changes following spinal manipulation in an ovine in vivo model with disc degeneration. Neurophysiological responses to spinal manipulative impulses (20 N, 40 N, 60 N, 80 N thrust magnitude and 10 ms, 100 ms, 200 ms thrust duration) were recorded to mimic device-assisted (<10 ms) and manually (100–200 ms) delivered spinal manipulation. In sheep with lumbar disc degeneration, there was a 20–25% reduction in positive EMG responses and a 4.5–10.2% increase in compound action potential instantaneous frequency response as a result of spinal manipulation compared to control. [21] In a human case study, in vivo intradiscal pressures (L3/L4) were measured in a healthy individual during a side-lying manually delivered spinal manipulation resulting in peak intradiscal pressures of 662 kPa over 290 ms (in the absence of spinal joint cavitation), and 890 kPa over 125 ms (with spinal joint cavitation) [23]. These peak intradiscal pressures were similar in magnitude to healthy study participants while sitting upright (550–623 kPa) or sitting in a flexed position (830–1133 kPa) [24,25]. However, due to the invasive nature of these types of physiological recording procedures, very few in vivo studies (in animals or humans) have measured deep tissue pressure changes during spinal manipulation. While spinal manipulative forces reaching deep spinal structures in cadaveric studies has been recently defined [11], specific attenuation of these manipulative forces in deep spinal tissues in vivo remain unclear. In a recent pilot study using a similar in vivo feline experimental preparation, Activator V spinal manipulation resulted in mean lumbar intradiscal pressure changes of ~2.8 kPa, ~2.9 kPa, ~2.6 kPa, and ~2.6 kPa for device settings 1–4, respectively [26]. Interestingly, intradiscal pressures remained relatively constant regardless of Activator device setting, which was consistent with data collected when using a separate commercially available spinal manipulation device (Pulstar) [26]. Increased knowledge and understanding of the deep spinal musculature response to spinal manipulation may eventually become a better indicator of treatment success compared to changes in intradiscal pressure as these deep spinal muscles represent the active movers of the spine, while the vertebrae and intervertebral discs are passive structures. Greater knowledge regarding preferential loading and/or deep spinal tissue physiological response to loading may help to illuminate some of the underlying physiological mechanisms of spinal manipulation. As an initial step in this direction, we used an established feline in vivo preparation to characterize in vivo superficial and deep intramuscular pressures changes related to the delivery of clinically relevant spinal manipulation thrust durations using a feedback motor (100 ms thrust duration) or electromagnetic solenoid-powered device (Activator V; <5 ms duration).

## 2. Materials and Methods

Animal protocols were reviewed and approved by the University of Alabama at Birmingham Institutional Animal Care and Use Committee (#21667) with spinal manipulation data being collected from 19 adult male (*n* = 11) and female (*n* = 8) feline preparations (4.2–5.8 kg). General surgical procedures related to this experimental preparation are described in detail elsewhere [27]. Briefly, anesthesia was induced with ketamine (5–10 mg/kg) and dexmedetomidine (10–20 µg/kg) and a peripheral saphenous IV catheter inserted for fluids and anesthesia (Nembutal; initial bolus of 10–35 mg/kg; supplemented by constant rate infusion (0.2–5 mg/Kg/h). Orotracheal intubation was performed, and a catheter was placed in the common carotid artery to monitor blood pressure and arterial gas levels throughout the experiment. Mechanical ventilation was provided throughout the experiment and upon completion of all facets of the study, the animal was euthanized with an overdose of pentobarbital.

Certain surgical procedures were performed to permit concurrent muscle spindle recordings in these experimental preparations as part of a separate study. The L5 vertebra was exposed by a left paraspinal incision and the right L6 dorsal rootlets were exposed by an L5 laminectomy (vertebra levels were confirmed upon completion of the terminal experiment). All L6-L7 paraspinal muscle tissues, articulating lumbar facet joints, and lumbar discs remained intact. For intramuscular recordings, miniature pressure catheters (Millar SPR-1000) were inserted via a stainless-steel cannula into the left paraspinal muscle above (superficial) and below (deep) to the L7 transverse process from the L5 exposed surgical site. Unlike the human spine with five lumbar vertebrae, the feline spine has seven lumbar vertebrae. The deeply anesthetized animal remained in the prone position for all spinal manipulation procedures. Stereotaxic equipment was used to stabilize the spine at the L4 spinous process (clamp) and ilium bilaterally (hip pins) for neurophysiological recordings.

### 2.1. Spinal Manipulation Devices

**Figure 1:**
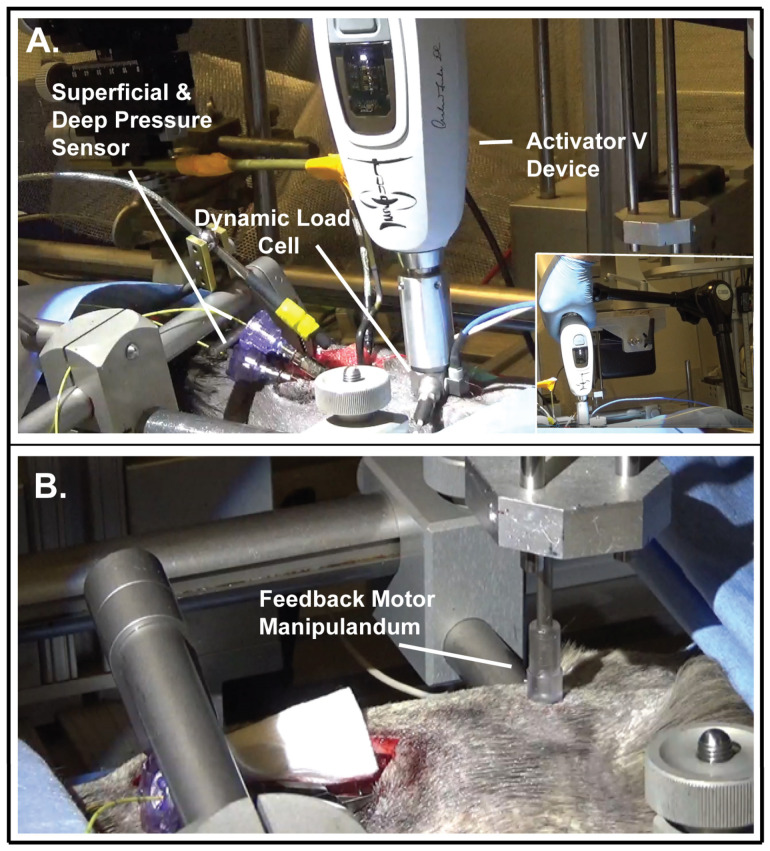
Experimental set-up. Photographs of (**A**) Activator V device with inset showing device being held by Noga articulated holder with the experimenter’s hand used only to trigger the device, (**B**) Feedback motor manipulandum (tip).

A commercially available clinical spinal manipulation device (Activator V; Activator Methods Int. Ltd., Phoenix, AZ, USA) was used to deliver extremely short thrust durations (2–3 ms) to the L7 spinous process in a posterior-to-anterior direction. The Activator V device has four progressive settings (1–4), all of which deliver a solenoid generated mechanical shockwave. It delivers up to a maximum mean peak force of 220 N when tested directly on a load cell and 52, 68, 112 and 155 N at settings 1 to 4 when tested against a stiff spinal tissue analog (258.07 N/mm) with handheld delivery [7]. Activator V device testing using a more compliant spinal tissue analog (30.22 N/mm) resulted in further attenuation of mean peak forces of 35, 63, 102 and 130 N at settings 1 to 4, respectively [7]. Specific attenuation of spinal manipulative forces in vivo remains unclear, particularly in deep paraspinal musculature. Prior to Activator V impulse delivery, a preload force (~2.9 N) is required which was accomplished by gently lowering the device (held by an articulated holder) onto the dorsal surface of the animal over the L7 spinous process (Figure 1A). The rubber tip of the clinical Activator V device was removed and a custom-made device sleeve with an impedance head that consisted of a dynamic load cell was attached to the Activator V device (Figure 1A). The diameter of the Activator V circular contact surface was 15.6 mm, equating to an area of 1.91 × 10^−4^ m^2^. To help standardize impulse delivery, the Activator V device was held via a Noga articulated holder and the only manual contact with the device was in relation to engaging the device trigger (Figure 1A inset). A period of 5 min separated successive impulses delivered at randomly selected device settings to mitigate viscoelastic change in the preparation [28]. Intramuscular pressure related to Activator V manipulative thrusts were recorded both in the absence and/or presence of muscle spindle recordings from teased dorsal root fibers (neurophysiological data not included). Computer generated simple randomization of Activator device settings 1–4 was used in the absence of dorsal root muscle spindle recordings, whereas similar randomization methods were used between device settings 1 and 2 when simultaneously recording muscle spindle afferent activity from finely teased dorsal nerve root filaments. This greater occurrence of lower device settings was done to avoid potential damage from higher Activator V device settings to the finely teased dorsal root filament which were wrapped around the recording electrode. Therefore, Activator V force and intramuscular pressure data was collected on device setting 1 (*n* = 33), setting 2 (*n* = 31), setting 3 (*n* = 15), and setting 4 (*n* = 15).

### 2.2. Feedback Motor Device

A programmable electronic feedback system (Aurora Scientific, Lever System 310) with displacement and force motor control was used to deliver spinal manipulation to the L7 spinous process in a posterior-to-anterior (vertical) direction at a 100 ms thrust duration. A detailed description of this feedback motor spinal manipulation device has been reported elsewhere [26,27]. Spinal manipulative thrusts were delivered under displacement control. Manipulative thrusts under both displacement and force control have been used in previous work in this feline model [27]. The feedback motor lever arm (with plexiglass manipulandum attached via a custom made rotary-to-linear converter) was programmed to advance at a rate of 0.5 mm/s to peak thrust target of either 1, 2, 3, 5, 8, or 10 mm and a thrust duration of 100 ms. The forces required to obtain the desired thrust displacements were recorded. The manipulandum was mechanically lowered until contact was made with the intact cutaneous tissue overlying the L7 spinous process (Figure 1B). For these particular experiments, no contact load or tissue preload was used prior to the manipulative thrust being delivered so as to better ascertain intramuscular pressure response and the role of paraspinal viscoelastic tissue compliance related strictly to short duration manipulative thrusts. A range of vertebral displacements from 1 to 10 mm was used to provide a more complete picture of intramuscular pressure change during spinal manipulation, and to ensure vertebral displacement had indeed occurred due to a lack of tissue preload prior to the manipulative thrust. The diameter of circular manipulandum tip was 7.22 mm (Figure 1B) equating to an area of 4.09 × 10^−5^ m^2^. Computer generated simple randomization of feedback motor displacement settings (1, 2, 3, 5, 8, 10 mm) were used in the absence of dorsal root muscle spindle recordings (*n* = 13), whereas as part of a separate muscle spindle study, a greater number of randomized 5, 8, and 10 mm manipulative thrust displacements were recorded at these displacements (*n* = 51).

### 2.3. Statistical Analysis 

All the outcomes were summarized as mean and standard deviation (SD). Between-group comparison was conducted using two sample t test for two groups. For more than two groups, the analysis of variance (ANOVA) followed by Tukey’s HSD test for multiple comparisons was used. The within-group comparison was conducted using paired t test. Pearson correlation analysis was used to explore the correlation among different outcomes. All the tests were two-sided at the significance level of 0.05, using SAS 9.4 (Cary, NC, USA). 

## 3. Results

**Figure 2:**
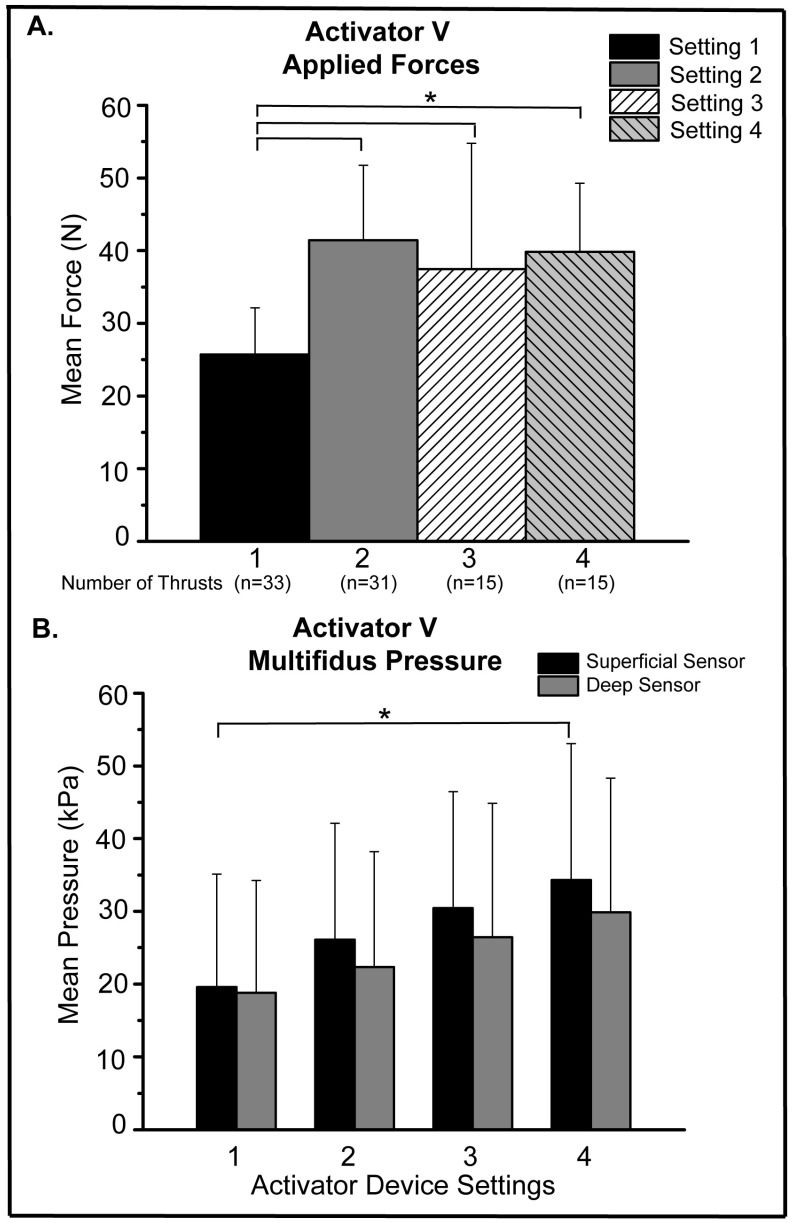
Activator V Applied Force and Intramuscular Pressure. (**A**) Mean Activator V L7 spinous applied forces at devices settings of 1–4, (* *p* ≤ 0.003); (**B**) mean superficial and deep intramuscular pressure at Activator V device settings 1–4 (* *p* = 0.024).

For Activator V spinal manipulation, there was a significant increase in mean applied forces in vivo when comparing forces delivered using the 1st device setting to forces delivered at the 2nd, 3rd, 4th device settings (Figure 2A; *p* ≤ 0.003). No other significant differences in mean applied forces were found between any other Activator V device settings, with device settings 2, 3, and 4 all delivering mean forces in vivo approximating 40 N (Figure 2A). Regarding intramuscular pressures, incremental pressure increases were seen at both superficial and deep multifidi sensors at successive Activator V device settings 1–4 (Figure 2B). However, the only significant difference among the superficial and/or deep sensors was the comparison between the 1st and 4th device setting at the superficial sensor level (Figure 2B; *p* = 0.024). For intramuscular pressure differences between superficial and deep sensors, there was a 4.1% and 12.8% decrease in mean pressure measured at the deep sensor compared to the superficial sensor at Activator V device settings 1 and 4, respectively. However, paired t-tests indicated the differences between superficial and deep mean intramuscular pressures were not statistically significant at any particular Activator device setting (Figure 2B). In addition, Pearson correlation analysis indicated a positive correlation between the Activator thrust force and superficial intramuscular sensor pressure (r = 0.31, *p* = 0.003), but not between the Activator thrust force and the deep intramuscular sensor pressure. 

**Figure 3:**
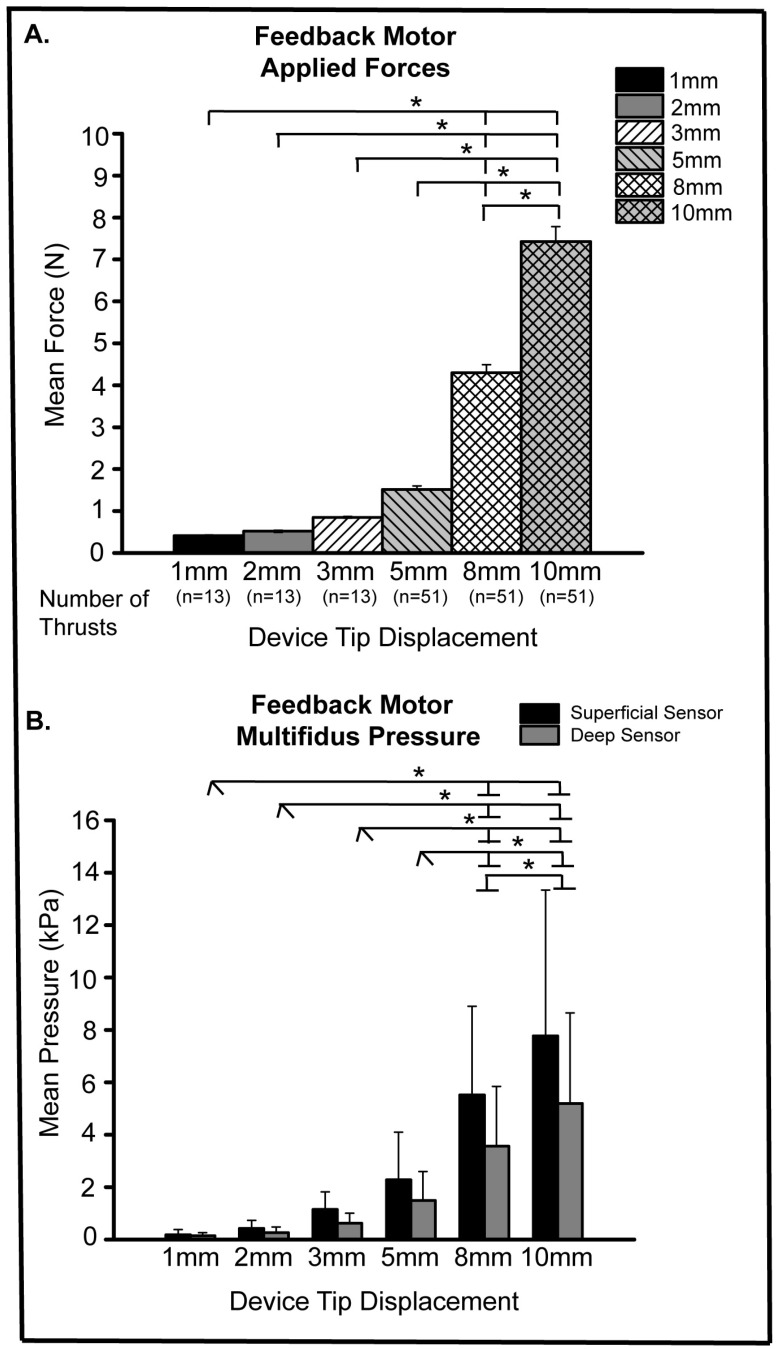
Feedback Motor Applied Force and Intramuscular Pressure. (**A**) Mean feedback motor applied forces for L7 spinal tissue displacements of 1, 2, 3, 5, 8, 10 mm (* *p* < 0.0001); (**B**) mean superficial and deep intramuscular pressures for L7 spinal tissue displacements of 1, 2, 3, 5, 8, 10 mm (* *p* ≤ 0.0002).

For the feedback motor manipulative thrusts, greater soft tissue/vertebra displacement resulted in increases in applied forces ranging from 0.4 N to 7.4 N for 1 mm and 10 mm, respectively (Figure 3A). Significant differences in feedback motor applied forces related to spinal tissue displacement are shown in Figure 3A, with a near exponential increase in applied forces with greater tissue displacement. It is noteworthy that mean feedback motor forces at the greatest displacement (10 mm) were 18–20% of the Activator V mean forces at device settings 2, 3, and 4. A stair-step increase in mean superficial and deep multifidus muscle pressures occurred with increasing applied feedback motor forces (Figure 3B). The 1 and 10 mm displacements yielded a 16.7% and 33.2% decrease, respectively in mean intramuscular pressure between the superficial and deep sensors. Unlike with the Activator V, feedback motor force which was delivered at a slower thrust rate was positively correlated with mean intramuscular pressure changes at both the superficial and deep intramuscular sensor.

## 4. Discussion

To our knowledge, this study is the first to record intramuscular pressure changes related to spinal manipulation delivered by a commercially available clinical device (Activator V) and a feedback motor in an in vivo animal model. This study demonstrates that in vivo soft tissues vastly dampen superficially applied manipulative forces which may have potential mechanistic, safety, and/or clinical implications. 

It is noteworthy that higher Activator V device settings did not coincide with incremental increases of applied forces in this in vivo model (Figure 2A). This suggests either an inherent capacity of in vivo soft tissues to similarly dampen manipulative forces within a certain range of thrust magnitude, or a possible indication that peak force alone may not be the best indicator of deep muscle tissue response. In vitro testing of the Activator V device revealed that it mimicked a half-sine thrust curve. However, that profile is slightly altered across the device settings due to the dynamics of the solenoid-ambos principle. Small changes in thrust profile can result in meaningful changes of the cumulative frequency spectrum of the thrust impulse. The fact that the mean muscle pressure response correlated with device setting indicated a positive correlation of yet unknown biomechanical factors. The current findings are supported by previous work in which spinal manipulative impulses delivered by two separate clinical manipulation devices (Pulstar and Activator V) yielded similar intradiscal pressure changes regardless of device setting [26]. The viscoelastic properties of soft tissues and/or the incompressibility of water in paraspinal soft tissues likely contributed to this plateau effect in intramuscular and intradiscal pressure related to short duration spinal manipulative thrusts. Compared to testing the Activator V device directly onto a load cell yielding mean peak forces up to 220 N, mean manipulative forces in vivo are substantially lower being less than 45 N. Similar diminution of total peak forces of 300 N were reported in a porcine cadaveric model, where only 12.1% of the total peak force reached deep spinal structures in the lumbar spine [11]. Additional support for this substantial degree of dampening of applied spinal manipulative forces is found when Activator V thrusts were delivered to spinal tissue analogs which yielded a ≤59% decrease in applied manipulative forces compared to when the device was tested directly onto a load cell [7]. In humans, the range of manipulative forces applied to the lumbosacral region is reported to be between 220–889 N [5,29,30,31], which brings the 26–41 N delivered in vivo by the Activator V into proper context. This variance in peak thrust force highlights the working principle of the Activator V device in that the device itself does not generate a force but an inertial motion of the device tip. In response, the tissue resists that motion which is then measured in terms of force. A high resistance or stiff tissue will experience a high thrust force while compliant or soft tissues will experience only a minor thrust force. Consequently, the thrust force generated has to be considered in context with the compliance or stiffness of the actual tissue that receives the treatment.

Another aspect to be considered is the remote location of the muscle response measurements compared to where the thrust is applied. A mechanical thrust applied to the spinous process propagates through the vertebra and the surrounding tissues, including the muscles. Viscoelastic properties of hard tissues (e.g., bone) are significantly different from those of soft tissues (e.g., muscles). The viscoelastic dampening and resonance frequencies generally do not overlap, meaning that an enhanced response for one tissue may result in significant dampening within the other. Future work should seek to include mathematical models and optimization routines investigating the optimal settings for manipulative thrust signal propagation in an effort to overcome such challenges.

Our current findings highlight the need to better define the role that viscoelastic and other soft tissue properties play with regard to in vivo dampening of spinal manipulative thrust forces. A better understanding of the relationship between superficially applied spinal manipulation forces and deep spinal tissue responses in vivo will help provide a more comprehensive picture of mechanoreceptor-related mechanisms that might be essential to positive clinical outcomes with spinal manipulation. As previously noted, the greatest increase in Activator V mean peak forces occurred between device settings 1 and 2 with nominal changes in in vivo force at subsequent device settings. That said, mean peak Activator V forces at any device setting were substantially greater (18–34%) than feedback motor applied spinal manipulative forces. Likewise, mean superficial and deep multifidi intramuscular changes were several times greater using the Activator V device compared to the feedback motor (even at the greatest feedback motor displacement of 10 mm).

Overall, the feedback motor generated forces were orders of magnitude smaller than those delivered using the Activator V device (Figure 3A). Mean peak forces increased near exponentially at larger displacements (5, 8, 10 mm) with similar pattens demonstrated among superficial and deep intramuscular pressure recordings. As expected and similar to the Activator V findings, feedback motor mean intramuscular pressures at the superficial multifidus sensor were greater than those at the deep sensor, but for the most part these pressure differences failed to reach significance with either spinal manipulation device. This suggests that small manipulative loads reach deep tissue structures to a similar degree as greater loads, but viscoelastic and other soft tissue properties act to dampen applied manipulative forces uniformly. It further indicates that peak thrust force may not be the ideal parameter to predict intramuscular response. Feedback motor forces were somewhat lower than those recorded in previous spinal manipulation experiments using displacement control [27]. However, this is most likely due to the absence of contact load or tissue preload in the current set of experiments. In past experiments, a high-resolution optical recording system was used to determine the forces required to actuate the vertebra for each animal, but vertebra actuation was not required to achieve the current objective of determining the impact of viscoelastic dampening of spinal manipulative in vivo forces. 

Limitations of this study include that intramuscular pressure was recorded in a deeply anesthetized animal model, thus normally experienced muscular reflexive responses that would occur in non-anesthetized preparations were absent. Presence of muscle reflexive responses would have only further enhanced the amount peak force dampening demonstrated presently with the animal under anesthesia. However, even with the presence of anesthesia, use of an in vivo preparation is of particular benefit as it provides insights to other viscoelastic tissue properties which are absent in cadaveric tissues. It is possible, but unlikely that use of stereotaxic spinal stabilization for a neurophysiological recording study could have influenced the lumbosacral tissues. The L4 spinous clamp and the hip pins placed on the external ilium were of sufficient distance away from the L7 spinous process and surrounding paraspinal tissues that any influence from spinal stereotaxic stabilization would have been minimal. While the intramuscular pressure is a direct measure of the manipulative thrust impact at the level of deep spinal musculature, it remains to be seen whether a particular intramuscular pressure is a prerequisite to any specific biological response in superficial or deep multifidi muscle tissue. This question will need to be answered in future in vivo research. 

## 5. Conclusions

This is the first study that reports in vivo changes in deep intramuscular pressure using a commercially available spinal manipulation device and/or a feedback motor. Peak spinal manipulation forces were markedly diminished due to viscoelastic properties of the surrounding in vivo spinal tissues. These findings demonstrate the need to further investigate and understand in vivo viscoelastic relationships between applied peak spinal manipulative forces and various physiological responses in deep spinal tissues such as the multifidus muscle and/or intervertebral discs. 

## Data Availability

The data presented in this study are available on reasonable request from the corresponding author. The data are not publicly available due to cybersecurity reasons.
